# Action Observation Plus Sonification. A Novel Therapeutic Protocol for Parkinson’s Patient with Freezing of Gait

**DOI:** 10.3389/fneur.2017.00723

**Published:** 2018-01-04

**Authors:** Susanna Mezzarobba, Michele Grassi, Lorella Pellegrini, Mauro Catalan, Bjorn Kruger, Giovanni Furlanis, Paolo Manganotti, Paolo Bernardis

**Affiliations:** ^1^Department of Life Sciences, University of Trieste, Trieste, Italy; ^2^Azienda Sanitaria Universitaria Integrata di Trieste, Trieste, Italy; ^3^Department of Medical, Surgical and Health Sciences, University of Trieste, Trieste, Italy; ^4^Gokhale Method Institute, Palo Alto, CA, United States

**Keywords:** freezing of gait, action observation, Sonification, Parkinson’s disease, cueing

## Abstract

Freezing of gait (FoG) is a disabling symptom associated with falls, with little or no responsiveness to pharmacological treatment. Current protocols used for rehabilitation are based on the use of external sensory cues. However, cued strategies might generate an important dependence on the environment. Teaching motor strategies without cues [i.e., action observation (AO) plus Sonification] could represent an alternative/innovative approach to rehabilitation that matters most on appropriate allocation of attention and lightening cognitive load. We aimed to test the effects of a novel experimental protocol to treat patients with Parkinson’s disease (PD) and FoG, using functional, and clinical scales. The experimental protocol was based on AO plus Sonification. 12 patients were treated with 8 motor gestures. They watched eight videos showing an actor performing the same eight gestures, and then tried to repeat each gesture. Each video was composed by images and sounds of the gestures. By means of the Sonification technique, the sounds of gestures were obtained by transforming kinematic data (velocity) recorded during gesture execution, into pitch variations. The same 8 motor gestures were also used in a second group of 10 patients; which were treated with a standard protocol based on a common sensory stimulation method. All patients were tested with functional and clinical scales before, after, at 1 month, and 3 months after the treatment. Data showed that the experimental protocol have positive effects on functional and clinical tests. In comparison with the baseline evaluations, significant performance improvements were seen in the NFOG questionnaire, and the UPDRS (parts II and III). Importantly, all these improvements were consistently observed at the end, 1 month, and 3 months after treatment. No improvement effects were found in the group of patients treated with the standard protocol. These data suggest that a multisensory approach based on AO plus Sonification, with the two stimuli semantically related, could help PD patients with FoG to relearn gait movements, to reduce freezing episodes, and that these effects could be prolonged over time.

## Introduction

For decades, motor and gait difficulties have been identified as the main symptoms of Parkinson’s disease (PD), and drug therapy—based on dopamine and its agonists—was considered the only feasible solution to ameliorate symptoms. Amid these motor symptoms and gait abnormalities, freezing of gait (FoG) is the most debilitating; a sudden episodic inability to generate an effective stepping, which commonly, leads to falls.

However, PD is a complex neurological disease that comprises also severe psychiatric and cognitive symptoms. Today, the benchmark to treat PD symptoms, especially when they worsen, is the use of specific rehabilitation protocols together with medication and/or surgical therapy.

Drug therapy in PD is a symptomatic therapy, primarily aimed at restoring dopaminergic function in the striatum. Although irreplaceable in the treatment of PD symptoms, several data demonstrate also negative effects, produced by dopamine on certain movements, and cognitive functions. Indeed, while dopaminergic medication clearly enhances certain motor functions, at the same time might negatively affect the learning of movement sequences (1, 2), as well as specific cognitive functions (3, 4). Moreover, the absent or controversial pharmacological responsiveness of FoG has led to an increasing interest in rehabilitation interventions aimed at functional recovery and autonomy, by relearning a physiological gait pattern.

Currently, protocols employed for rehabilitation of PD—with and without FoG—are based on the use of external sensory cues—mainly visual, but also auditory and tactile—because it allows the switch from *automatic* movement (habitual)—controlled by frontostriatal pathways, that PD patients have compromised—to *voluntary controlled* movement [goal directed (5)]. Specifically, Vandenbossche et al. (6) showed that PD patients with FoG exhibit a specific impairment in the acquisition of automaticity—correlated with the working memory functions—and suggested that therapies should focus on training that reduce working memory load, as the cued strategies.

During exposure to visual and auditory cues, patients with FoG, as those without, improve gait kinematics and reduce freezing. Interestingly, visual cues have more powerful effects than auditory cues for reducing FoG (7); proving that the inability to maintain effective scaling of step amplitude could be an important FOG-related deficit. Conversely, auditory cues (metronome) seem to be less effective in the regularization of altered cadence, and disordered coordination of inter-limb movement in patients with FoG. Unfortunately, it has been shown that cueing might generate an important dependence on the environment, particularly the visual ones, considering how important is the exploration of the whole visual field in intentional walk (8).

In the last years, several researchers try to use cues differently. Young et al. (9) asked Parkinson’s patients with and without FoG to listen to different auditory cues (i.e., a metronome or ecological footsteps sounds recorded on gravel), and to step in place to each cue, synchronizing their own stepping in time to the sound. Results in patients with FoG showed remarkable improvements in temporal regularity. The authors claim that in PD patients with FoG, the mechanism “action imitation enhances the motor performance” is supported by their results with action-relevant cues (i.e., footsteps recorded on gravel).

Teaching *motor strategies*, without cues to overcome or avoid freezing episodes can be an alternative/innovative approach to rehabilitation, that matters most on appropriate allocation of attention (10), and lightening cognitive load. One of these strategies—action observation (AO)—is based on the activation/sharing of a common neural substrate, the mirror system (11). The *priming effect* of AO on subsequent motor execution of the observed gesture is well known in neurorehabilitation, although few evidences are available for treatment of patients of PD (12).

Furthermore, one way to reduce cognitive load in the recovery/learning of motor gestures is the use of multisensory approaches that enhance perceptual processes (13), which are known to be reduced in PD patients with FoG (14). The use of multisensory stimuli improves the learning process (13, 15) thanks to a reduced cognitive load, and to an easier storage in short-term memory (16, 17). But, to exert the most efficient facilitatory effect, pairs of stimuli composing the multisensory stimulus should be congruent, and not simply concomitant in space and/or time (18, 19). These findings have stimulated interest toward the use of audiovisual stimuli to facilitate relearning of movements also in the field of neurological rehabilitation.

Evidence on the efficacy of action-related *sonified* sounds (synthetized sounds obtained with a *Sonification* procedure, see the next paragraph) to improve motor performance is well documented [for a review, see Ref. (20)], although in PD patients is still limited. Indeed, Rodger et al. (21) used two different types of sounds (ecological and synthetized) to help guide and improve walking actions of PD patients. One of these techniques was based on Sonification of the ground reaction forces. Both methods showed that PD patients could use rich auditory representations of action to guide and improve the quality of walking, and reducing the risk of falls and injury. Moreover, Schmitz et al. (17) demonstrated that the Sonification of movements enhance the activity in the human AO system including subcortical structures of the motor loop; and therefore, may be an important method to enhance therapy effects in neurological rehabilitation.

The most natural way to use audio–video stimuli is to present images together with *ecological* sounds (i.e., a walker and the sound of his/her feet). Instead of utilizing the real sounds produced during gait, we employed synthetized sounds obtained with the Sonification technique (22). Specifically, in our audiovisual stimuli, the auditory component is obtained by transforming kinematic data of relevant body part movements—visible in the video—into sounds. This process is called *Sonification*. We choose to use sonified sounds—in place of real sounds (i.e., footsteps sound)—because in this way we can convey additional information, important for the understanding and reproduction of a correct movement (i.e., differences in the velocity of the hips rotation during gait), that otherwise will be ignored. This final stimulus is a sort of *augmented* audio–video stimulus. The processing of auditory and visual information together facilitates the recognition of the movement in its spatial and temporal aspects, and the relearning process of the correct pattern of movements. These stimuli could be of particular importance for PD patients with FoG in which these components are altered, and in which, probably, visuo-perceptive modifications may be present (14).

We hypothesized that AO can be used to facilitate recovery of defective motor control, and given that PD patients with FoG may have major shortages of attention resources, a multisensory approach (i.e., audiovisual stimuli) would help to further reduce the attention load, facilitating learning processes.

The aim of this study was to test the efficacy of a novel protocol based on AO technique and Sonification, and to compare the effect with a standard protocol based on external sensory cues. With this purpose, we designed and realized an experimental study to test the effectiveness of these two protocols in two groups of PD patients with FoG. We hypothesized that gait improvement of the AO plus Sonification protocol would be better than those obtained with the standard protocol, both in the short term and the long term (3-month follow-up).

## Materials and Methods

### Design

The whole pilot RCT was carried out from April 2015 to December 2016. Post-intervention measures were collected at the end, 1 month, and 3 months after the end of the treatment. Patients were randomly assigned to two different training group (experimental and control groups). An investigator, neither involved in the treatment protocol nor in the selection and evaluation of patients, created the computerized randomization procedure (blocked randomization). The same investigator concealed treatment allocation by using small opaque envelopes. Three trained physical therapists with a solid experience in the treatment of PD were involved in the evaluation—one of them—and in the treatment of patients—the other two. Outcome measures were videotaped and also evaluated by a second independent rater blind to the whole experimental study. In case of discrepancies between the two, a third blind rater was used to resolve the evaluation.

Patients were advised to have their medical treatment continued unchanged throughout the study. This study was carried out in accordance with the recommendations of the “Comitato Etico Regionale Unico” guidelines, with written informed consent from all subjects. All subjects gave written informed consent in accordance with the Declaration of Helsinki. The protocol was approved by the Institutional Ethics Committee (Comitato Etico Regionale Unico—Friuli Venezia Giulia. Protocol no. 4456—05.02.2015). Patients who agreed to participate always signed a written informed consent and they were able to leave the experiment at any moment, with no additional explanations. The study has been registered at http://Clinicaltrials.gov, NCT03249155.

### Participants

**Figure 1:**
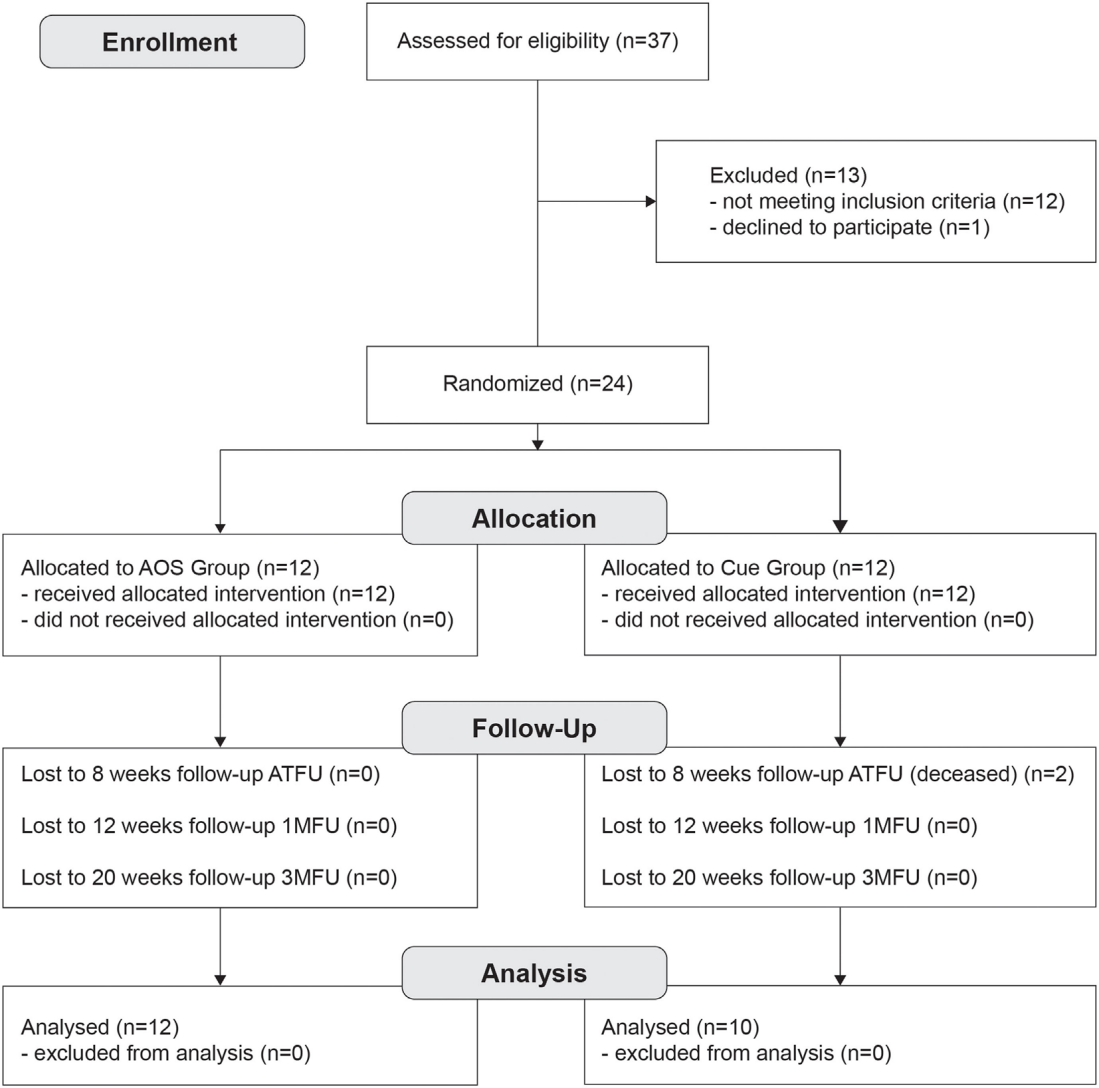
Flowchart showing the structure of the study, enrollment and evaluation procedure, and how the patients were divided into the groups, and phases.

Thirty-seven patients with idiopathic PD (see Figure 1), according UK Brain Bank (23) were assessed by a neurologist expert in movement disorders, from the outpatient Neurological Clinic, Cattinara Hospital. Eligibility criteria were occurrence of FoG (24) based on patient’s verbal account of his/her freezing experience (or recognition of their typical FoG experience when this symptom was described to him/her by a physician); stages 1–3 on the Hoehn and Yahr scale (25); stable medication regimen for at least 8 weeks; no major depressive symptoms as defined by a Beck Depression Inventory score ≤16 [BDI (26)]; no signs of dementia as defined by a Mini-Mental Status Examination score >24 [MMSE (27)]. The exclusion criteria were evidence of any adjunctive orthopedic comorbidities that make it impossible to use physical activities and an independent locomotion; others neurological and psychiatric disease; presence of any implanted stimulating or pacing device in central nervous system. Prior power analysis estimated a sample size group of 10 participants. After the first assessment, we enrolled a total of 24 patients (see Figure 1). Two subjects dropped out due to concurrent, unrelated medical events: thus, 22 patients completed the study (see Table 1).

**Table 1:** Demographic and clinical characteristics of patients with Parkinson’s disease at baseline.

	AOS group (*n* = 12)	Cue group (*n* = 10)
**Patients’ characteristics**		
Gender female/male (% female)	5/7 (42%)	3/7 (30%)
Age (years)	74.67 ± 5.93	72 ± 5.87
Disease duration (years)	10.75 ± 3.44	9.4 ± 4.86
Mini-Mental State Exam	27.46 ± 1.81	26.58 ± 1.1
Beck Depression Inventory	8 ± 5.98	6.4 ± 5.93
Hoehn and Yahr stage	2.33 ± 0.49	2.3 ± 0.67
Levodopa equivalent	972.5 ± 253.17	983.22 ± 379.58
**Primary outcome measure**		
NFOGQ	18.17 ± 4.61	16.6 ± 7.86
**Secondary outcome measures**		
UPDRS II Total	16.42 ± 5.99	17.1 ± 6.47
UPDRS III Total	32.92 ± 8.69	33.2 ± 13.99
MPAS	53.75 ± 6.27	50.2 ± 9.22
BBS	47.75 ± 4.16	47.8 ± 3.46
6MWT (s)	280.75 ± 93.34	296.75 ± 48.32
TUG (ms)	1,299.75 ± 376.74	1,271.3 ± 615.86
PDQ39 mobility	49.38 ± 22.54	35.5 ± 25.87
PDQ39 activities of daily living	33.4 ± 21.99	28.75 ± 17.84
PDQ39 emotional well-being	23.82 ± 21.84	33.33 ± 20.13
PDQ39 stigma	21.04 ± 22.94	18.75 ± 20.41
PDQ39 social support	2.78 ± 6.49	15.83 ± 22.03
PDQ39 cognitions	23.23 ± 15.52	31.88 ± 19.64
PDQ39 communication	24.31 ± 20.86	29.17 ± 21.25
PDQ39 bodily discomfort	31.67 ± 21.92	27.5 ± 19.27
PDQ39 Total	51.67 ± 26.9	50.8 ± 29.43

*Data are mean ± SD or as otherwise indicated*.

*n, number of patients; NFOGQ, New Freezing of Gait Questionnaire; UPDRS, Unified Parkinson’s Disease Rating Scale II and III; PDQ39, Parkinson’s disease questionnaire 39; MPAS, Modified Parkinson’s Activity Scale; BBS, Berg Balance Scale; 6MWT, 6-min walking test; TUG, Time-Up-and-Go; PDQ39, 39-item PD Questionnaire*.

### Experimental Procedures

All participants underwent to a 1 h of rehabilitation training during their ON condition (approximately 1 h after the antiparkinsonian medication intake), twice a week, for 8 consecutive weeks, and a total of 16 training sessions.

#### Experimental Group

The protocol was based on the AO method plus Sonification (AOS). During each training session, eight videos, showing an actor performing eight different motor gestures, were presented to the patient that then tried to repeat, according to the Modeling principles (28). Each video, lasting 1.5 min, was composed by images (from fronto-lateral perspectives) and sounds (obtained with Sonification) of eight specific motor gestures. These gait related gestures were useful for ameliorating weight shifting, step scaling, and bilateral coordination of stepping, known as locomotion features related to FoG. In each session, all videos were presented, from simple to complex motor actions. The contents of the eight videos are reported in Appendix. Each session started with the observation of the audio–video projected on a large sized screen (2.5 m × 2 m) located in front of the patient at a distance of 2 m. During AO, to increase the accuracy of imitation, patients were asked to attend to the peculiar characteristics of the observed action, and no movements were allowed. At the beginning, after video observation, patients had to practice repetitively the observed actions for the same time (1.5 min). Then, patients performed *on line* the same motor gesture while they were watching the videos. With the aim to facilitate the modeling process, an expert physiotherapist in AO treatment, encouraged and corrected patient’s motor execution. Each video was repeated twice.

#### Control Group

The same eight motor gestures were performed also in the Cue control group with the same order and amount of time, by using attentional strategies. During each training session, patients were asked to practice the motor gesture by means of visual (stripes on the floor) or auditory (metronome) cues, to facilitate the learning of temporal and spatial parameters. As for the experimental group, the expert physiotherapist encouraged and corrected each patient’s motor execution to facilitate correct motor learning process. Following physical therapist’s instructions, patients progressively learned to perform the eight motor gestures without cues.

Participants of both groups were instructed to not practice further rehabilitation/physiotherapy treatments during the duration of the study. The two therapists involved in the treatments were not dedicated to one group, but equally assigned to both of them.

### Clinical Outcomes

The patients who met inclusion/exclusion criteria underwent to a clinical and motor functional evaluation before the treatment (BT), after the treatment (AT), 1 month (1MFU), and 3 months AT (3MFU). The neuropsychological evaluations were only done at the baseline and 1 month (1MFU) AT, since the minimum interval for test administration is 3 months. All clinical evaluations were performed by an experienced neurologist, and a physiotherapist blinded to participants’ allocation.

As primary outcome, FOG duration and severity were assessed by using New Freezing of Gait Questionnaire (NFOGQ). Particularly, we calculate an index of improvement obtained at AT, 1MFU, and 3MFU evaluations, respect to the BT evaluation (see Data Analysis).

*A priori* power analyses based on a previous experiment that compared the two treatment protocols in individuals with PD and FoG (29), suggested 10 participants per group to achieve a medium effect size (*f* = 0.45, alpha *p* = 0.05, power = 0.95, critical *F* = 4.41). We recruited 12 participants for each group to account for possible attenuation.

As for the secondary outcomes, disease severity was tested with the Unified Parkinson’s Disease Rating Scale (UPDRS II–III), the Hoehn and Yahr scale (25), and quality of life with the 39-item PD Questionnaire (30). Motor functional performance evaluation included Modified Parkinson’s Activity Scale (31), Timed Up and Go (32), and 6-min walking test (33). Berg Balance Scale (34) was used to assess static and dynamic balance capabilities. Also for the secondary outcome measures, we calculated an improvement index.

#### Neuropsychological Evaluation

We assessed patients’ most important cognitive functions, useful for learning new motor ability: executive functions, attention, and memory capabilities (Table 2), to exclude that the cognitive profile of the patients of each group was changed after the end of the treatments. This was important to exclude that different levels of efficacy were due to differences in the cognitive profile of the two groups of patients. Global cognitive functioning was tested with the Montreal Cognitive Assessment (35); short-term and long-term memory functions with Digit Span backward (36), Corsi Test (37), Babcock Story Recall Test (38); attention with the Attentive Matrices (39), the Stroop Test (40), and Trail Making Test: parts A and B (41). Executive functions were evaluated with the Frontal Assessment Battery (42) and Tower of London Test (43). Abstract reasoning by Raven matrices (44). All neuropsychological tests scores were corrected on age, sex, and education using normative values. Moreover, patients were always tested in “ON” condition during their optimal antiparkinsonian medication.

**Table 2:** Cognitive profile of patients with Parkinson’s disease at baseline and at 1-month follow-up (1MFU).

Cognitive domain	Test	AOS group at baseline	Cue group at baseline	AOS group at 1MFU	Cue group at 1MFU

		Mean ± SD	Mean ± SD	Mean ± SD	Mean ± SD
Short-term memory	Digit Span Backward	4.84 ± 1.27	4.84 ± 1.14	4.59 ± 1.14	4.58 ± 1.02
	Corsi Test	4.85 ± 0.84	4.34 ± 0.84	4.50 ± 0.83	5.01 ± 1.44

Long-term memory	Babcock Story Recall Test				
	Immediate recall	5.78 ± 1.51	4.52 ± 2.48	5.27 ± 1.62	3.83 ± 2.50
	Delayed recall	5.8 ± 1.4	4.41 ± 3.07	5.16 ± 1.88	3.41 ± 3.27

Attention	Attentive Matrices	40.94 ± 8.35	32.46 ± 12.86	36.91 ± 11.27	31.56 ± 13.51
	Trail Making Test:				
	Part A (s)	30.35 ± 21.59	68.51 ± 46.78	52.44 ± 45.98	55.27 ± 38.02
	Part B (s)	147.34 ± 116.51	190.58 ± 130.99	148.35 ± 199.33	166.68 ± 117.38
	Stroop Test				
	Time (s)	28.78 ± 19.19	52.73 ± 42.05	42.59 ± 26.17	47.72 ± 29.42
	Errors	3.01 ± 3.55	1.83 ± 1.76	3.64 ± 2.47	3.36 ± 3.39

Executive functions	Frontal Assessment Battery	16.7 ± 1.22	16.19 ± 1.71	16.55 ± 1.78	16.11 ± 1.80
	Tower of London Test	17.99 ± 1.4	19.56 ± 6.38	18.61 ± 0.29	18.22 ± 1.41

Reasoning	Raven’s progressive matrices	29.71 ± 6.18	27.28 ± 5.17	28.85 ± 6.16	27.70 ± 5.31

*Data are mean ± SD or as otherwise indicated*.

### Audiovisual Stimuli

The short video used in the experimental group showed two healthy actors’ (one male and one female) performing the 8 motor gestures from a lateral and frontal perspective, for a total of 32 different videos (2 gender × 8 gestures × 2 perspectives). Moreover, prior rehabilitation treatment, and to be comfortable with the procedures, each participant practiced the tasks using other videos showing three movements test. The sounds of each video were obtained with the Sonification technique, by transforming kinematic data (i.e., velocity) recorded during the execution of the eight gestures, into audio pitch variations. Actors performed all tasks barefoot, walking along a 10-m walkway surrounded by a seven-camera motion-capture Qualisys System (120 Hz). During the execution of each motor gestures, kinematic data were collected recording four retroflective markers placed on the left and right anterior superior iliac spine to calculate pelvis movement velocity, and on the left and right lateral malleoli to calculate inferior limbs velocity. All data were recorded and preprocessed by a dedicated software Qualisys Track Manager, and frame by frame instantaneous speed was obtained, and transformed into pitch audio by the open source framework Pd (45) using modules developed by Henkelmann and colleagues (46, 47). The Sonification itself is done in the following steps: first, median filter with a window size of three frames is applied to the kinematic data to suppress sensor noise from the kinematic data. Second, the data stream is linearly scaled to an interval from 0 to 1. Third, the pitch sound itself is generated. Forth, the sound is mapped to the left or right audio channel. The PD module we used to generate our stimuli can be found in the supplemental materials for this publication. 32 audio track were gained (2 actors gender × 8 different gestures × 2 perspectives) for each motor gestures, and 2 audio tracks for each movement test. Videos were edited by using Final Cut Pro X software, with a dubbing procedure to merge the sounds with the video part of each gesture. The kinematic–acoustic recording was provided with a visual auditory stimulus congruence.

### Data Analysis

Preliminary, applying the Kolmogorov–Smirnov (KS) test we verified the sustainability of a normal distribution for the primary and secondary clinical outcomes. Highly skewed and kurtotic variables were log transformed and then KS tested for the effectiveness of the correction. Outcomes that failed this second test where excluded from the analysis.

For each clinical outcome, the improvement (gain) from pretraining to posttraining (AT, 1MFU, and 3MFU) was computed for each participant by subtracting each person’s pretraining score from his/her posttraining score and dividing the difference for the pretraining performance. Formally:

$$\text{gain}=\frac{\text{post-training}-\text{pre-training}}{\text{pre-training}}.$$

Systematic differences in pretraining scores between the two groups of patients were preliminary excluded with *t*-tests on both primary and secondary outcomes measures. As for the cognitive profile, we verified for potential differences in the pretraining (BT) and modification after 3 months (1MFU) with a 2 × 2 mixed factors ANOVA (Group and Time of evaluation). The results are reported in Table 3.

**Table 3:** Split plot ANOVA results.

	SS	Mean sq	F	Pr(>F)	η^2^G (%)
**Main effect of treatment (Group)**					

**Primary outcome measure**					
NFOGQ	2.741	2.741	24.28	0.000	50
**Secondary outcome measures**					
PDQ39 mobility	12.630	12.633	14.91	0.001	38
UPDRSIII	4.962	4.962	13.36	0.002	33
PDQ39 bodily discomfort	4.962	4.962	13.36	0.002	33
PDQ39 Total	2.031	2.031	9.73	0.006	27
UPDRSII	1.427	1.427	11.14	0.003	24
BBS	0.121	0.121	6.42	0.020	24
6MWT	0.470	0.470	4.11	ns	15
TUG	1.070	1.071	3.95	ns	14
MPAS	0.021	0.021	0.56	ns	2
PDQ39 cognitions	0.471	0.471	0.47	ns	2

**Main effect Time (within subjects)**					

**Primary outcome measure**					
NFOGQ	0.091	0.046	3.11	ns	3
**Secondary outcome measures**					
PDQ39 mobility	0.151	0.075	0.60	ns	1
UPDRSIII	0.334	0.167	2.14	ns	3
PDQ39 bodily discomfort	0.334	0.167	2.14	ns	3
PDQ39 Total	0.158	0.079	2.05	ns	3
UPDRSII	0.049	0.025	0.48	ns	1
BBS	0.005	0.003	3.01	ns	1
6MWT	0.061	0.031	2.91	ns	2
TUG	0.222	0.111	3.66	0.035	3
MPAS	0.009	0.005	0.69	ns	1
PDQ39 cognitions	1.546	0.773	4.17	0.023	6

**Interaction effect (Time × Group)**					

**Primary outcome measure**					
NFOGQ	0.002	0.001	0.07	ns	0
**Secondary outcome measures**					
PDQ39 mobility	0.386	0.193	1.54	ns	2
UPDRSIII	0.197	0.098	1.26	ns	2
PDQ39 bodily discomfort	0.197	0.098	1.26	ns	2
PDQ39 Total	0.261	0.131	3.41	0.043	5
UPDRSII	0.047	0.023	0.46	ns	1
BBS	0.002	0.001	0.90	ns	0
6MWT	0.022	0.011	1.03	ns	1
TUG	0.034	0.017	0.56	ns	1
MPAS	0.014	0.007	1.02	ns	1
PDQ39 cognitions	0.451	0.225	1.22	ns	2

*ns, not significant; NFOGQ, New Freezing of Gait Questionnaire; UPDRS, Unified Parkinson’s Disease Rating Scale II and III; PDQ39: Parkinson’s disease questionnaire 39; MPAS, Modified Parkinson’s Activity Scale; BBS, Berg Balance Scale; 6MWT, 6-min walking test; TUG, Time-Up-and-Go; PDQ39, 39-item PD Questionnaire; SS, sum of squares*.

*UPDRSIII, TUG, PDQ39 mobility, and PDQ39 bodily discomfort were log transformed for normality. Index η^2^G is the generalized eta-squared statistics calculated following the guidelines by Olejnik and Algina (48) and Bakeman (49). For the two main effects and interaction tested we used the formulas SSA/(SSA + SSs/A + SSPs/A), SSP/(SSP + SSs/A + SSPs/A) and SSPA/(SSPA + SSs/A + SSPs/A), where A and P refer to our variables Group and Time, respectively, and s represents the subject factor. Errors related SS are not shown in this table*.

The hypothesis of differences in improvement (gain) between the experimental and control groups was tested by a mixed design ANOVA on the gain scores using *Group* (AOS vs. Cue) as a between subjects factor, and *Time of evaluation* as within-subject factor. Besides main effects, we also considered the interaction terms *Group* × *Time* to assess the stability of the effect across evaluation. *Post hoc* Bonferroni’s test was employed to assess gain score differences between groups at each time. The significant change threshold was set at *p* ≤ 0.05. We interpreted the meaningfulness of the significant changes using the generalized eta-squared (η^2^G) statistics calculated following the guidelines by Olejnik and Algina (48) and Bakeman (49).

#### Linear Discriminant Analysis (LDA)

Besides these statistical criteria, to examine the clinical impact of the AOS and Cue training on outcomes scores, we used also an automated classification rates criterion. This method is commonly used as a technique for pattern classification. In our case, we used this method to compare or classify the clinical profiles of the patients in the two groups, and at the different stages of the experimental study. Automated classification problems involve continuous input variables (i.e., our clinical scales), and categorical outcomes (i.e., the rehabilitation protocol or the stage of the study). The algorithm has to learn to predict the category from the input data. We used an LDA algorithm in two differ ways, to discriminate between groups and within subjects. Finally, we choose to complement standard analysis of variance with this LDA because of recommendations on using simulative approaches to data analysis with small samples (50).

First, the algorithm learned between-groups’ discriminative criterion on a fraction of 70% of the data set, then for testing we applied the criterion on the remaining fraction of 30% (see Supplementary Material), measuring the classification accuracy in terms of sensitivity index (51). Average sensitivities were based on a complete random design, simulating all possible combination of 70–30% of the participants (50). Particularly, we trained and tested the LDA four times, over each evaluation time (BT, AT, 1MFU, and 3MFU), and considering the rehabilitation protocol attended by participants as the categorical outcome to predict.

Second, to evaluate stability over time, as in the case of interaction term in the ANOVA, we considered within-subject evaluations, using the LDA algorithm on a subset composed by 70% of pre- and posttraining individual’s outcomes, and testing it on the remaining 30% of pre- and posttraining individual’s outcomes.

In both cases, we expect that the more effective is the training in transforming the participants clinical profile, the more accurate is the LDA algorithm in (learn to) classify the participants within the training actually practiced. In the first LDA implementation, we expect that the LDA classification would fail only in the comparison between the two groups at the BT time window.

All the analyses were programmed using R statistical language (52).

## Results

At the baseline, there were no significant differences between groups with respect to demographics and clinical records, as shown in Table 1. Also for the cognitive profile, as reported in Table 2, there was no differences except for the interaction Group × Time in the Corsi test [*F*_(1,20)_ = 5.975, *p* = 0.024, η^2^ = 0.225], but when we compared the two groups in the two moments with a *t*-test, the difference was not significant [*t*(20) = 1.449, *p* = 0.163; *t*(20) = −0.480, *p* = 0.636].

### Primary Outcome Measure

**Figure 2:**
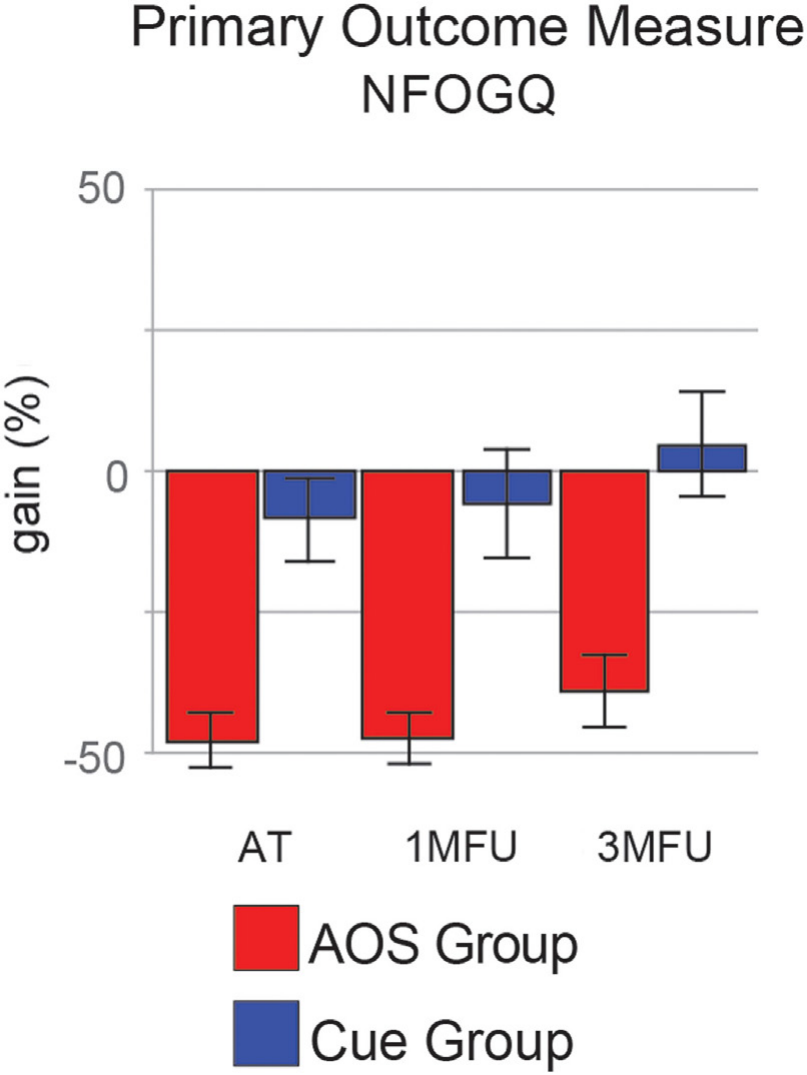
Improvement (gain) of the two groups in the primary outcome measure [New Freezing of Gait Questionnaire (NFOGQ)], at the three evaluation times. Error bars are 1 SE.

Action observation plus Sonification treatment had a significant positive effect in reducing the primary outcome measure, participant’s ratings of FoG severity and duration, as shown at the end of the treatment, and most important, at the second follow-up (Figure 2, NFOGQ). Noteworthy, on our sample the standard Cue protocol did not show any relevant gain effect from the baseline evaluation.

### Secondary Outcome Measures

**Figure 3:**
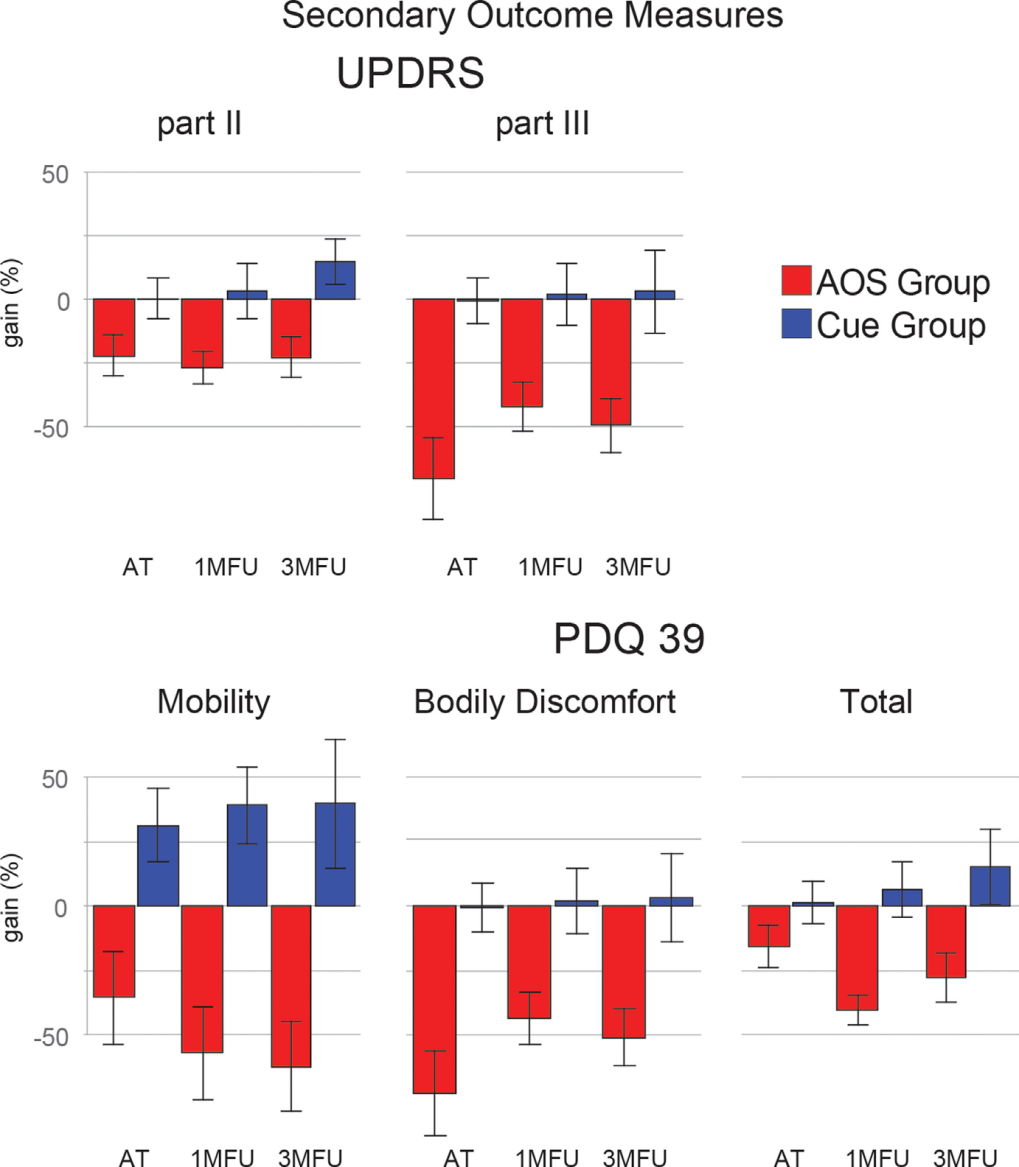
Improvement (gain) of the two groups in five secondary outcome measures [Unified Parkinson’s Disease Rating Scale: parts II and III (UPDRS) and Parkinson’s disease questionnaire 39 (PDQ39): mobility, bodily discomfort, and the total score], at the three evaluation times. Error bars are 1 SE.

Secondary outcome measures that improved in AOS (Figure 3) were as follows: severity of motor impairment (UPDRS III); motor problems, and bodily discomfort in activity of daily life (the mobility and bodily discomfort subscales of the PDQ39 questionnaire). For this pool of outcome measures, the positive effect of AOS treatment over Cue training has a great effect size (η^2^G > 0.30) and is stable until the last follow-up (see *post hoc* comparisons reported in Table 4). Even in these measures, the standard Cue protocol did not show any relevant gain effect. Figures 2 and 3 show the gain scores of the main effects.

**Table 4:** Bonferroni *post hoc* comparisons AOS group vs. cue group.

	After treatment	1st Follow-up	2nd Follow-up
**Primary outcome measure**			
NFOGQ	*p* ≤ 0.001	*p* ≤ 0.001	*p* ≤ 0.001
**Secondary outcome measures**			
PDQ39 mobility	*p* ≤ 0.05	*p* ≤ 0.001	*p* ≤ 0.001
UPDRSIII	*p* ≤ 0.001	*p* ≤ 0.05	*p* ≤ 0.05
PDQ39 bodily discomfort	*p* ≤ 0.001	*p* ≤ 0.05	*p* ≤ 0.05
PDQ39 Total	ns	*p* ≤ 0.01	*p* ≤ 0.01
UPDRSII	ns	*p* ≤ 0.05	*p* ≤ 0.01
BBS	ns	*p* ≤ 0.05	ns
6MWT	ns	ns	*p* ≤ 0.05
TUG	ns	ns	ns
MPAS	ns	ns	ns
PDQ39 cognitions	ns	ns	ns

*Data are p-levels of the direct comparisons at each evaluation time, between groups, after the Bonferroni correction*.

*NFOGQ, New Freezing of Gait Questionnaire; UPDRS, Unified Parkinson’s Disease Rating Scale II and III; PDQ39, Parkinson’s disease questionnaire 39; MPAS, Modified Parkinson’s Activity Scale; BBS, Berg Balance Scale; 6MWT, 6-min walking test; TUG, Time-Up-and-Go; ns, not significant*.

Table 3 reports *F*-tests for main effects and interaction separately, for all outcomes considered, whereas Table 4 reports direct comparisons at each evaluation time, between groups.

At first glance, for nearly all secondary outcomes, the group factor (the main effect of rehabilitation protocol) had the greater effect size (η^2^G). These effects are stable over time since interaction terms are not significant and/or with negligible amounts of variance explained.

The problems in activity of daily living (PDQ39 total score, UPDRS II) were significantly reduced by AOS training, with stable results also after 3 months. Moreover, AOS training determined also a small improvement on average gain scores of motor balance (BBS, see Tables 3 and 4).

### Linear Discriminant Analysis

**Figure 4:**
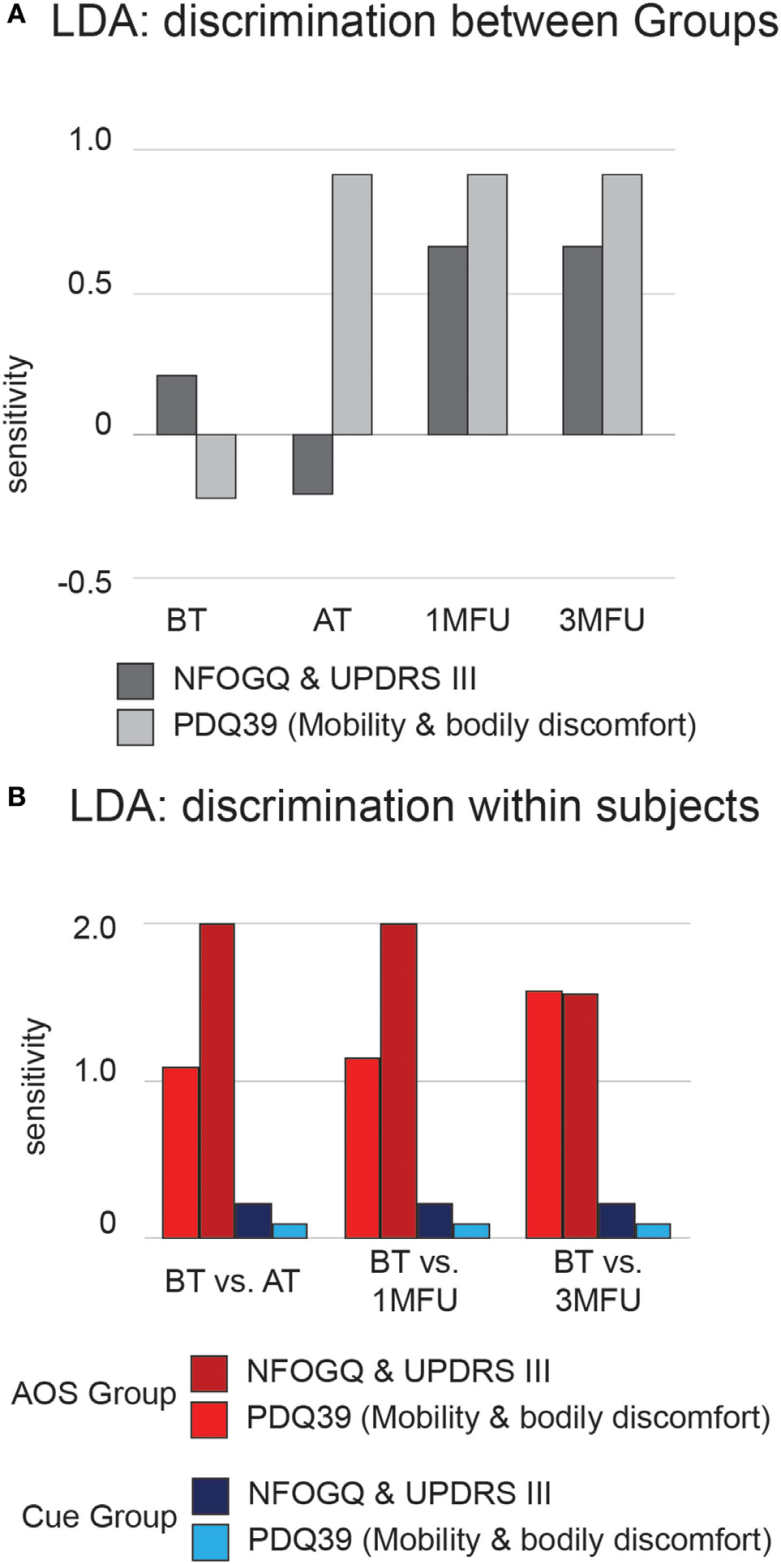
Average discrimination accuracy expressed in terms of sensitivity (*d*′) of the linear discriminant analysis (LDA) algorithm. **(A)** Results of testing between-groups’ discrimination. Dark gray bars represent LDA results from the New Freezing of Gait Questionnaire (NFOGQ) and UPDRS III scales. Light gray bars represent LDA results from the PDQ39 mobility and bodily discomfort scales. **(B)** Results of testing within-subject discrimination. Dark and light red bars represent LDA results from the NFOGQ and UPDRS III scales for the Action Observation plus Sonification experimental group. Dark and light blue bars represent LDA results from the PDQ39 mobility and bodily discomfort scales, for the Cue control group.

We trained an LDA algorithm to learn to discriminate between rehabilitation protocols attended by participants, using as input variables significant outcomes identified by ANOVA. Particularly, inclusion criteria were the following: (a) significant main effect on group factor and great effect size (>0.30), (b) stable result over evaluation time (no interaction in Table 1 and significant *post hoc* comparisons in Table 3). Input variables were the raw data, not transformed into gain scores. Figure 4A shows average discrimination accuracy, expressed in terms of sensitivity—i.e., SDs from chance (51). At the baseline, the algorithm could not learn a reliable criterion to recognize “AOS” or “Cue” participants since their clinical profiles are homogeneous (Table 2) and hence its performance stops to a chance level. Immediately AT, at 1 month, and after 3 months, experimental protocol differentiates participant’s outcomes from the baseline levels and the algorithm can learn a criterion that move the performance (nearly) 1 SD from the chance. Importantly, the effect is far more evident considering the mobility outcome (NFOGQ and UPDRS III), over the improvement in ability of daily activities (PDQ39 mobility and bodily discomfort).

Furthermore, using the same input variables, we trained an LDA algorithm to discriminate each participant’s pre- and posttraining conditions, within each group; Figure 4B shows average sensitivity index, separately for the AOS and Cue conditions. Participants trained with experimental AOS protocol were discriminable with respect to their baseline condition to an extent of 2 SD from chance, using clinical motor profile, and this result is quite stable over time. The ability of the algorithm to learn systematically a criterion over the chance was not proven, within participant’s undergoing rehabilitation with Cue.

## Discussion

Sonification and AO are used together for the first time with the aim to treat motor diseases in PD patients with FoG. The main finding of our study is that this multisensory treatment reduces FoG (number of episodes and duration), and provided positive effects on gait pattern in short- and long-term period.

These results are in agreement with those obtained in two previous studies with the use of AO: Pelosin et al. (29) and Agosta et al. (53). In both these studies, freezing improvements (assessed with FOGQ and NFOGQ, respectively) were evaluated only up to 1 month after the end of the treatment—instead of three—and with mixed and weak results—Pelosin’s data showed a significant improvement at the 1-month follow-up, but not at the end of the treatment, and the reverse pattern in the Agosta’s. Our data with AO plus Sonification showed consistent and significant effects in several of the secondary outcome measures: on motor impairment (UPDRS III), and quality of life (PDQ39 mobility scale), during the entire 3-month period of evaluation; while balance (BBS), gait parameters (6MWT), only at the first and second follow-up, respectively. Overall, our data confirmed the therapeutic potential of a protocol based on AO plus Sonification in treating gait disorders and FoG. In AOS group, patients improved their mobility, acquiring new motor strategies to overcome FoG, and these effects are prolonged over time and generalized to FoG in daily life.

In Cue control group, no enhancements were found for all mobility indices, throughout the three testing times. Only data in PDQ39 questionnaire subitem mobility and activities daily living show trend values toward an improvement, that with a larger sample, could lead to significance. A possible explanation is a residual cue dependence effect, which may have not triggered an effective learning process (54), and pointing out that evidences on the effectiveness of cue trainings in the alleviation of FoG symptoms is still a hot topic. Another potential factor could be the age of our sample. Although the two groups were not statistically different in age and stage of the disease, overall our patients were quite old (73 years). Probably, older patients may require a more specific training to engage a motor consolidation process when a standard protocol based on external sensory cues is used. Indeed, it should be emphasized the lower mean age of participants with PD and FoG in previous studies [66 years—Agosta et al. (53); 66 years—Lu et al. (55); 62 years—Young et al. (9)], when compared with the ones of our research. This age difference could have produced an additional decline in motor learning (56). In a crossover design with old patients with PD and FoG (mean age, 74 years), Bunting-Perry et al. (57)—using a laser beam on a rolling walker as a visual cue—showed no significant effects in diminishing FoG and improving walking.

The peculiar feature and novelty of our approach is the inclusion of a sonified audio track—representing kinematic features of a movement—to the video of the same movement. In other words, we used the Sonification to highlight task-intrinsic (spatial and temporal) information, otherwise difficult to access. This *augmented* stimulus is very different from those typically employed in AO treatments, since usually the sound part is absent (29), or not related in meaning to the content of the video [not congruent multisensory stimulus as in Ref. (53)]. When a patient attends to a stimulus with a sound cue (i.e., metronome) presented together with a video of an action, the amount of cognitive resources necessary to integrate the information of the two stimuli—not related in meaning—increase [for a review, see Ref. (58)]. In our protocol, the videos are congruent multisensory stimuli, in the sense that sounds and images are related in meaning, and probably bound together at the perceptual level. This conclusion is based on patients’ personal reports; they all reported to perceive stimuli as being highly consistent, and treated them as a single audiovisual event [the unity assumption—for a recent review, see Ref. (59)]. In fact, during the training, we did not need to use any particular type of instruction—except that the sound simply derived from the velocity of the movement—to let patient understand the meaning of sonified sounds, and the relation between the two sources of information (sound and image). After the presentation of the examples, the meaning of the stimuli became clear to almost all patients, and for those with some doubts, the presentation of the first stimulus was sufficient to understand.

The observation of action activates in humans the mirror neuron system (MNS) within the premotor cortex, inferior frontal gyrus, and inferior parietal lobule, that maps sensory signal onto the same neural circuits involved in motor planning and execution of the observed motor gesture. Congruent Sonification may have improved AO priming effect on movement. Indeed, during congruent audiovisual stimuli observation, Schmitz et al. (17) demonstrated an amplified activation of some of the major MNS areas, particularly frontal operculum, inferior parietal lobule and the superior temporal areas. Thanks to an enhanced perceptual analysis of the movement, congruent Sonification could lead to an improved neural representation of the observed motor action, and to an easier learning process thanks to a lightened cognitive load.

Motor learning involves the interaction of several components (60): extraction and processing of task-relevant sensory information, making decision aimed to define which movements to perform (and in which order), activating control processes, and finally a reactive and biomechanical control. A multisensory AO plus Sonification protocol might have aided the first phase of motor learning, facilitating the extraction and integration of visual and coherent auditory inputs, for a better understanding of spatial and temporal features of motor action.

Moreover, we hypothesize that our multisensory protocol—based on congruent and unitary stimuli—could have produced positive effects on memory, and specifically working memory processes. In fact, Lehmann and Murray (15) showed that semantically congruent multisensory stimuli can enhance subsequent processing and memory performance, and more recently Brunetti et al. (61) demonstrated that crossmodal correspondence (i.e., audiovisual congruent stimuli) produced faster reaction times and higher accuracy in a classical working memory task (*n*-back task). Given these findings, and given that PD patients are known be impaired in working memory processes [see, for example, Ref. (62)], the use of multisensory stimuli could have facilitated the processing and consequently the production of more effective gait patterns.

Sonification, as alternative, can be used in a rehabilitation program for patient with PD by generating additional real-time movement information, being suitable for integration with visual and proprioceptive perceptual feedback, while the patient is performing physical exercises. With ongoing training activity, synchronously processed auditory information should be initially integrated into the emerging internal models, enhancing the efficacy of motor learning. This is achieved by a direct mapping of kinematic and dynamic motion parameters to electronic sounds, resulting in continuous auditory and convergent audiovisual or audio-proprioceptive stimulus arrays.

A critical analysis of protocols’ features emphasize that learning strategies used in the two groups could be also partially different in terms of learning mechanisms. Indeed, they are more related to a modeling process—with movement-related analogic representations—in the experimental group, while in the control group followed a cueing approach—with abstract and propositional representations. Agosta et al. (53) used a similar experimental design with a control group that underwent to a motor learning process by instructions and an experimental group that improved motor action by AO. In our control group, we used visual and auditory cue whose effectiveness had already been stressed by several studies in PD patients with FoG, and these results could be also considered as a further confirmation of the effectiveness of the AO therapy.

The combination in our AO plus Sonification protocol of a multisensory and analogic approach instead of a unisensory and abstract approach, produced promising positive effects, although we cannot evaluate nor the relative impact of each component, neither the effect of their interaction. However, this matter remains to be fully address.

Finally, as for the long-lasting effects, our protocol was a not intensive 8-week training program, which is not a long rehabilitative period from a motor learning and physical exercise perspective. Nevertheless, given that our results showed both immediate (upon the end of treatment), and long-term retention (3 months following cessation of treatment) of gait improvement, we may suppose that these benefits can be explained with a neuroplasticity process induced by goal-based exercises (63). As reported in previous studies (63, 64), goal-based exercise can promote neuroplasticity effects, which have been demonstrated in several neurological conditions, and also in PD, through changes in cortical excitability and cortical representation. Recently, using fMRI, Agosta et al. (53) demonstrated AO-related performance enhancement in patients with PD and FoG was possible with an intensive 4-week training program (12 sessions) and was associated with an increased activation of motor cortical areas and fronto parietal regions of the MNS.

## Ethics Statement

This study was carried out in accordance with the recommendations of the “Comitato Etico Regionale Unico” guidelines, with written informed consent from all subjects. All subjects gave written informed consent in accordance with the Declaration of Helsinki. The protocol was approved by the Institutional Ethics Committee (Comitato Etico Regionale Unico—Friuli Venezia Giulia. Protocol no. 4456—05.02.2015). Patients who agreed to participate always signed a written informed consent and they were able to leave the experiment at any moment, with no additional explanations. The study has been registered at http://Clinicaltrials.gov, NCT03249155.

## Author Contributions

Conception, and design of the research project: SM and PB. Organization of the research project: SM, PB, and PM. Execution of the research project: SM, LP, MC, BK, GF, and PB. Treatment of the patients: SM and LP. Statistical analysis and interpretation of data; writing of the manuscript first draft: SM, MG, and PB. Manuscript review and critique: SM, MG, LP, MC, BK, PM, and PB.

## Conflict of Interest Statement

The authors declare that the research was conducted in the absence of any commercial or financial relationships that could be construed as a potential conflict of interest.

## Appendix

### Exercise 1

Shifting the body weight in the frontal plane and taking a step—Actor stands straight up, with both feet on the floor, shifting the body weight to the right (or to the left), to the left (or to the right), and then raise and move forward the right (or the left) leg and the body to take the first step.

### Exercise 2

Shifting the body weight in the sagittal plane and taking a step—Actor stands straight up with both feet on the floor. One foot placed in front of the other, with the heel ahead of the other foot’s toes. The actor shifts weight from one foot to the other, always keeping the feet on the floor; afterwards he takes a step forward.

### Exercise 3

Gait initiation—Actor starts to walk with the preferred leg.

### Exercise 4

Turning around—Actor walks two steps with a straight trajectory, and then made a 180° turn in a narrow quarter (U-turn).

### Exercise 5

Stepping over an obstacle—Actor walks three steps with a straight trajectory, and then steps over the obstacle (obstacle’s height: 10% of patient’s height).

### Exercise 6

Sit-to-walk—Actor is seated on a backless and armless stool (knee angle 100°), and then raises and walks three steps forward.

### Exercise 7

Walking straight with long steps—Actor walks about 10 long steps with a straight trajectory trying to maintain a steady pace and to take long steps.

### Exercise 8

Walking through a doorway—Actor walks three steps with a straight trajectory, moves through a real doorway without stopping and then continue to walk two more steps.
