# Supplementary material for: Action Observation Plus Sonification. A Novel Therapeutic Protocol for Parkinson’s Patient with Freezing of Gait
Source: Front Neurol. 2018 Jan 4;8:723. doi: 10.3389/fneur.2017.00723 (PMC5758544; doi:10.3389/fneur.2017.00723)
Supplement: Supplementary file 1 [file Table_1.DOCX]

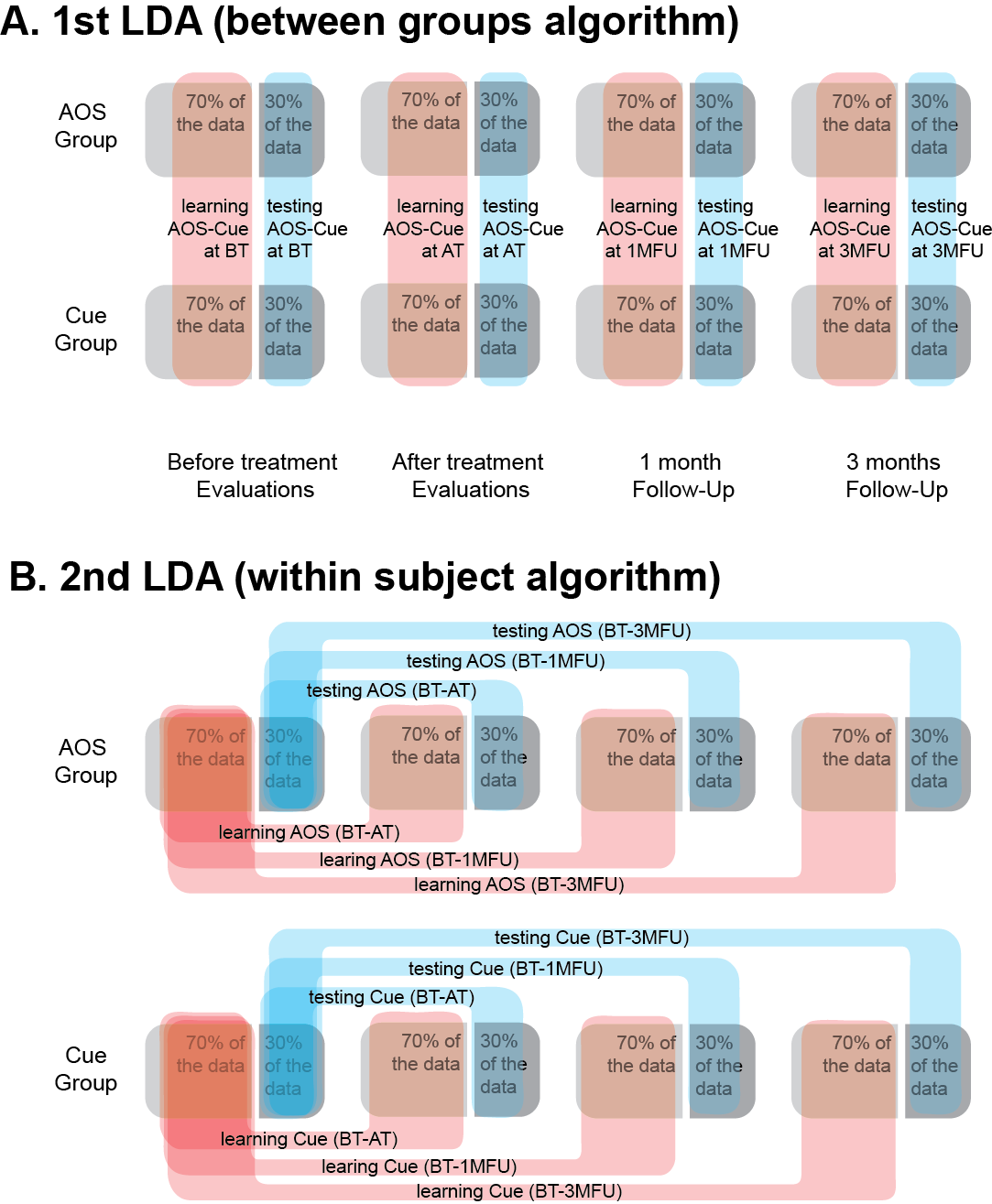


Light and dark grey boxes represent 70% and 30% of data collected during the evaluation of each group in the 4 time windows (before treatment, after treatment, 1-month follow-up, and 3-months follow-up). Red and light-blue transparent bands indicate how the data were put together for the learning phase (in red) and the following testing phase. Panel A represent the method used in the 1^st^ LDA, testing for between groups differences. Panel B represent the method used in the 2^nd^ LDA, testing for within subject differences.
